# GABAergic and Cortical and Subcortical Glutamatergic Axon Terminals Contain CB_1_ Cannabinoid Receptors in the Ventromedial Nucleus of the Hypothalamus

**DOI:** 10.1371/journal.pone.0026167

**Published:** 2011-10-11

**Authors:** Leire Reguero, Nagore Puente, Izaskun Elezgarai, Juan Mendizabal-Zubiaga, Miren Josune Canduela, Ianire Buceta, Almudena Ramos, Juan Suárez, Fernando Rodríguez de Fonseca, Giovanni Marsicano, Pedro Grandes

**Affiliations:** 1 Department of Neurosciences, Faculty of Medicine and Dentistry, Basque Country University, Leioa, Spain; 2 Fundación IMABIS, Málaga, Spain; 3 “Endocannabinoids and Neuroadaptation”, INSERM U862 NeuroCentre Magendie, Université Bordeaux 2, Bordeaux, France; Institute for Interdisciplinary Neuroscience, France

## Abstract

**Background:**

Type-1 cannabinoid receptors (CB_1_R) are enriched in the hypothalamus, particularly in the ventromedial hypothalamic nucleus (VMH) that participates in homeostatic and behavioral functions including food intake. Although CB_1_R activation modulates excitatory and inhibitory synaptic transmission in the brain, CB_1_R contribution to the molecular architecture of the excitatory and inhibitory synaptic terminals in the VMH is not known. Therefore, the aim of this study was to investigate the precise subcellular distribution of CB_1_R in the VMH to better understand the modulation exerted by the endocannabinoid system on the complex brain circuitries converging into this nucleus.

**Methodology/Principal Findings:**

Light and electron microscopy techniques were used to analyze CB_1_R distribution in the VMH of *CB_1_R*-WT, *CB_1_R*-KO and conditional mutant mice bearing a selective deletion of CB_1_R in cortical glutamatergic (Glu-*CB_1_R*-KO) or GABAergic neurons (GABA-*CB_1_R*-KO). At light microscopy, CB_1_R immunolabeling was observed in the VMH of *CB_1_R*-WT and Glu-*CB_1_R*-KO animals, being remarkably reduced in GABA-*CB_1_R*-KO mice. In the electron microscope, CB_1_R appeared in membranes of both glutamatergic and GABAergic terminals/preterminals. There was no significant difference in the percentage of CB_1_R immunopositive profiles and CB_1_R density in terminals making asymmetric or symmetric synapses in *CB_1_R*-WT mice. Furthermore, the proportion of CB_1_R immunopositive terminals/preterminals in *CB_1_R*-WT and Glu-*CB_1_R*-KO mice was reduced in GABA-*CB_1_R*-KO mutants. CB_1_R density was similar in all animal conditions. Finally, the percentage of CB_1_R labeled boutons making asymmetric synapses slightly decreased in Glu-*CB_1_R*-KO mutants relative to *CB_1_R*-WT mice, indicating that CB_1_R was distributed in cortical and subcortical excitatory synaptic terminals.

**Conclusions/Significance:**

Our anatomical results support the idea that the VMH is a relevant hub candidate in the endocannabinoid-mediated modulation of the excitatory and inhibitory neurotransmission of cortical and subcortical pathways regulating essential hypothalamic functions for the individual's survival such as the feeding behavior.

## Introduction

The hypothalamus plays a crucial role in regulating energy balance and food intake [1]. The ventromedial nucleus (VMH) is placed in the tuberal region of the hypothalamus and is associated with several homeostatic and behavioral functions, including regulation of appetite, energy balance, sexual behavior, anxiety, thermogenesis, cardiovascular functions and pain [2], [3]. Functionally, the dorsomedial VMH participates in the regulation of energy homeostasis, whereas the ventrolateral VMH controls female reproduction [2], [4].

While the large majority of VMH neurons expresses abundant vesicular glutamate transporter VGluT2 mRNA [5]–[7], only weak GAD65 mRNA and GAD67 mRNA signals are observed in this nucleus [6], [7]. The VMH is surrounded by hypothalamic GABAergic neurons [8] and about 12% of the ventrolateral VMH neurons are GABAergic [6].

The VMH has been proposed as a satiety nucleus that provides a strong excitatory input to arcuate neurons, contributing to the activation of anorexigenic neuronal pathways [9], [10]. The endocannabinoid system is implicated in endocrine regulation and energy balance. The derivatives of *Cannabis sativa* are well known to regulate food intake and the endocannabinoid system controls neuronal signaling in hypothalamic networks [11]. Although low levels of cannabinoid receptors are present in the hypothalamic nuclei [12], [13], their efficiency is higher than in other brain regions [14]. Moreover, specific cannabinoid receptor binding is found in several hypothalamic areas, including the VMH, which also expresses high levels of CB_1_R mRNA [13]. Although the overall CB_1_R immunolabeling intensity is much lower in the hypothalamus than in other brain regions, the VMH, in particular, exhibits a moderate CB_1_R immunostaining [15].

The levels of the two main endocannabinoids, anandamide and 2-arachydonoylglycerol (2-AG), in the hypothalamus are higher during fasting and lower following food intake reaching a critical point that favors a motivational state for food intake [11], [16]–[18]. The administration of anandamide into the VMH also stimulates appetite in rats [19]. In contrast, both chronically-treated animals with CB_1_R antagonists [11], [20], [21] and CB_1_R null mice [11], [20], [22] display an anorexigenic phenotype. Furthermore, activation of presynaptic CB_1_R inhibits the excitatory and inhibitory neurotransmission in neuronal circuits involved in eating behaviors [11], [18], [23]–[25]. Indeed, Glu-*CB_1_R*-KO conditional mice that do not express CB_1_R in neurons of cortical origin exhibit a hypophagic phenotype after food deprivation very similar to the full *CB_1_R*-KO. On the contrary, GABA-*CB_1_R*-KO mutants that lack CB_1_R in forebrain GABAergic neurons are hyperphagic under the same experimental conditions [26].

Taken together, it is well established that the endocannabinoid system exerts a neuronal modulation through the activation of presynaptic CB_1_R localized on both excitatory and inhibitory pathways in distinct brain networks regulating homeostatic and behavioral functions including food intake. In view of the described observations that both the endocannabinoid system and the VMH play a role in ingestive behaviors, the aim of this study was to analyze the CB_1_R contribution to the molecular architecture of the excitatory and inhibitory synaptic terminals in the mouse VMH. For this purpose, preembedding immunocytochemical techniques for light and high resolution electron microscopy were used. Highly specific CB_1_R antibodies were applied to the VMH of conditional mutant mice with a selective deletion of CB_1_R mainly from cortical glutamatergic (Glu-*CB_1_R*-KO) and mainly from forebrain GABAergic neurons (GABA-*CB_1_R*-KO) [27], [28]. Mutants with the lack of CB_1_R in all the cells of the body (*CB_1_R*-KO mice) were also examined [29].

## Results

### Immunolocalization of CB_1_R in the VMH

**Figure 1:**
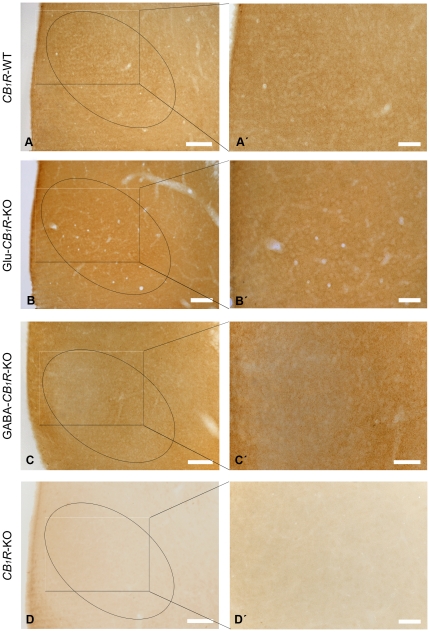
CB_1_R immunostaining in the mouse VMH. Preembedding immunoperoxidase method for light microscopy. VMH (oval circle in A-D) shows a moderate punctate CB_1_R staining in *CB_1_R*-WT (A, A') and Glu-*CB_1_R*-KO (B, B') mice. However, the CB_1_R immunoreaction product decreases in the VMH of GABA-*CB_1_R*-KO mice, particularly in the dorsomedial part (C, C'). The CB_1_R pattern disappears in *CB_1_R*-KO tissue (D, D'). Framed areas in A, B, C, D are enlarged in A', B', C', D'. Scale bars: 100 µm (A, B, C, D), 50 µm (A', B', C', D').

In the light microscope, the CB_1_R immunoreactivity was uniformly distributed throughout the entire VMH of *CB_1_R*-WT (Fig. 1A) with a somehow similar appearance in the Glu-*CB_1_R*-KO mice (Fig. 1B). At higher magnification, the pattern consisted of abundant small immunoreactive dots densely packed within the oval-shaped VMH (Fig. 1A', B'). However, CB_1_R staining decreased drastically in the VMH of GABA-*CB_1_R*-KO mice (Fig. 1C), particularly in the dorsomedial part (Fig. 1C'), suggestive of the presence of CB_1_R in GABAergic profiles. The immunolabeling fully disappeared in the VMH of *CB_1_R*-KO mice (Fig. 1D, D').

**Figure 2:**
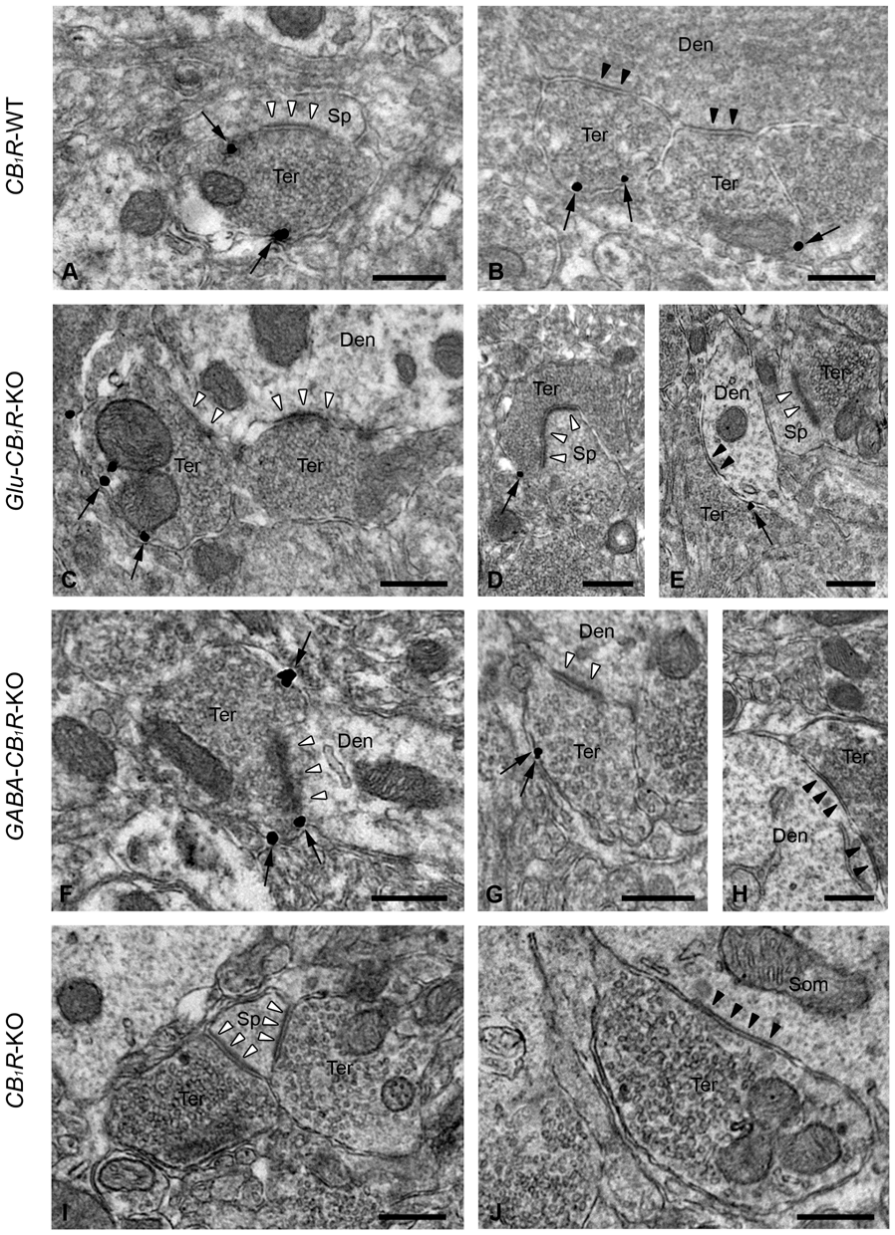
Ultrastructural localization of CB_1_R in the mouse VMH. Preembedding immunogold method for electron microscopy. A, B: In *CB_1_R*-WT, CB_1_R immunoparticles (arrows) are localized on membranes of presynaptic terminals (Ter) making asymmetric (white arrowheads) and symmetric synapses (black arrowheads) with dendritic spines (Sp) or dendrites (Den). C-E: In Glu-*CB_1_R*-KO, CB_1_R immunoparticles (arrows) localize to asymmetric synaptic terminals (Ter) presumably of excitatory subcortical neurons (observe the thick postsynaptic density marked with white arrowheads in D) as well as in inhibitory terminals (Ter) with symmetric synapses (black arrowheads in E). Notice CB_1_R immunonegative axon terminals (Ter) establishing asymmetric synapses (white arrowheads in C, E) with a dendrite (Den) or a spine (Sp). F-H: In GABA-*CB_1_R*-KO, CB_1_R immunolabeling (arrows) is in excitatory synaptic terminals (Ter) (see asymmetric synapses with white arrowheads in F and G) impinging on dendritic elements (Den). Observe in H a CB_1_R immunonegative synaptic terminal (Ter) making a symmetric synapse (black arrowheads) with a dendrite (Den). I, J: CB_1_R immunolabeling disappears in *CB_1_R*-KO mice indicating the specificity of the CB_1_R antibody used in the study. Note CB_1_R immunonegative synaptic terminals (Ter) making asymmetric (white arrowheads in I) and symmetric (black arrowheads in J) synapses with a dendritic spine (Sp) and a soma (Som), respectively. Scale bars: 0.4 µm.

**Figure 3:**
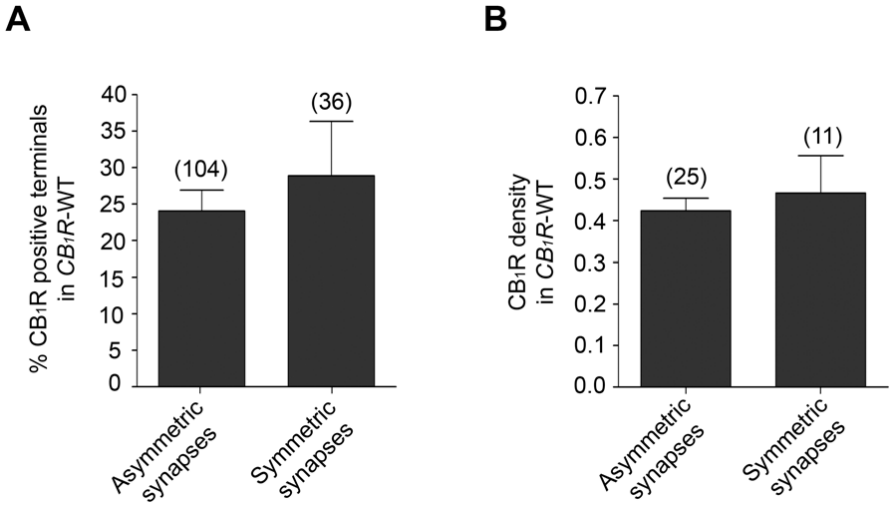
Statistical analysis of CB_1_R in terminals forming asymmetric and symmetric synapses in the VMH of *CB_1_R*-WT mice processed by a preembedding immunogold method. A: 24.0±2.9% of synaptic terminals making asymmetric and 28.9±7.5% of terminals with symmetric synapses are CB_1_R immunopositive. No statistically significant difference is detected (x^2^ = 0.5946, p = 0.4406, analyzed area: 2,376 µm^2^). B: CB_1_R density after subtraction of background labeling (0.015±0.003 particles/ µm in the VMH of *CB_1_R*-KO) is pretty similar in synaptic terminals making asymmetric or symmetric synapses (0.42±0.03 and 0.47±0.09 immunoparticles/ µm respectively, p = 0.6553).

Then, we analyzed the ultrastructural distribution of CB_1_R in the dorsomedial region of the VMH using a preembedding immunogold method for electron microscopy (Fig. 2). CB_1_R immunoparticles were typically localized away from the active zones on preterminal or synaptic terminal membranes making synapses with dendrites or dendritic spines. They showed characteristic features of excitatory (asymmetric synapses with obvious postsynaptic densities, abundant clear and spherical synaptic vesicles) and inhibitory (symmetric synapses with more pleomorphic synaptic vesicles) synapses (Fig. 2A, B). 24.0±2.9% and 28.9±7.5% of the synaptic terminals making asymmetric and symmetric synapses, respectively, were CB_1_R immunopositive in the VMH of *CB_1_R*-WT mice (Fig. 3A). In this case, CB_1_R density was 0.42 immunoparticles/ µm membrane in terminals making asymmetric synapses and 0.47 immunoparticles/ µm in terminals making symmetric synapses (Fig. 3B). There were no statistically significant differences in these parameters between terminals with asymmetric or symmetric synapses in the *CB_1_R*-WT mice.

To define the contribution of cortical glutamatergic and GABAergic synaptic terminals to the intrinsic CB_1_R pattern in the VMH, conditional CB_1_R mutant mice lacking the receptor either in cortical glutamatergic (Glu-*CB_1_R*-KO) or in forebrain GABAergic neurons (GABA-*CB_1_R*-KO) were used. CB_1_R was still observed in VMH axon terminals making synapses with dendritic and spiny elements of both mutant strains (Fig. 2C-H). In Glu-*CB_1_R*-KO mice, CB_1_R immunopositive terminals made asymmetric (Fig. 2C, D) and symmetric synapses (Fig. 2E). Also, CB_1_R immunonegative asymmetric synaptic terminals were found in the Glu-*CB_1_R*-KO mutants (Fig. 2C, E), suggesting the presence of CB_1_R in cortically-derived axon terminals. In GABA-*CB_1_R*-KO tissue, CB_1_R immunoparticles decorated presynaptic membrane profiles forming asymmetric (Fig. 2F, G) but not symmetric synapses (Fig. 2H). The immunolabeling was specific as the CB_1_R pattern disappeared in the VMH of *CB_1_R*-KO mice (Fig. 2I, J).

**Figure 4:**
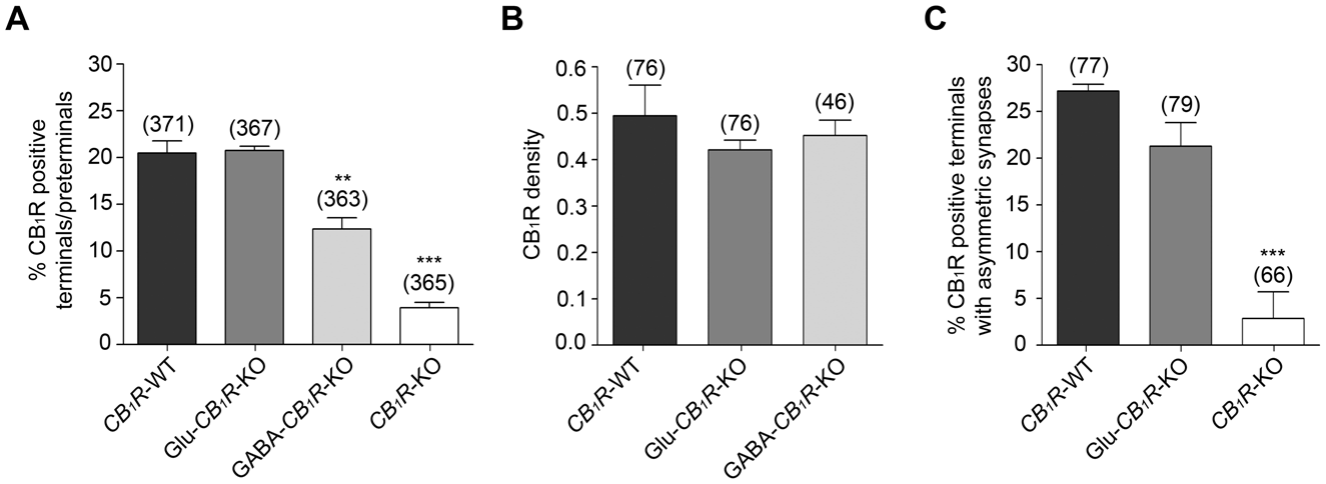
Statistical analysis of CB_1_R in the mouse VMH processed by a preembedding immunogold method. A: 20.5±1.3% of the synaptic terminals/preterminals are CB_1_R immunopositive in *CB_1_R*-WT mice. Similar proportion is in Glu-*CB_1_R*-KO (20.8±0.5%, x^2^ = 0.00024, p = 0.9876), lower in GABA-*CB_1_R*-KO (12.4±1.2%, x^2^ = 8.593, p = 0.0034) and virtually disappears in *CB_1_R*-KO mice (3.9±0.6%, x^2^ = 48.61, p<0.0001). A similar area was analyzed for each animal condition (1,467 µm^2^ in *CB_1_R*-WT; 1,562 µm^2^ in Glu-*CB_1_R*-KO; 1,646 µm^2^ in GABA-*CB_1_R*-KO and 1,519 µm^2^ in *CB_1_R*-KO mice). B: CB_1_R immunoparticle density after subtraction of background labeling (0.015±0.003 particles/ µm in the VMH of *CB_1_R*-KO) is very close in *CB_1_R*-WT (0.49±0.07), Glu-*CB_1_R*-KO (0.42±0.02, P = 0.7000) and GABA-*CB_1_R*-KO (0.45±0.03, P = 0.7000) mice. C: There is no statistically significant difference between the percentage of CB_1_R immunopositive asymmetric synapses in the VMH of *CB_1_R*-WT (27.2±0.7%) and Glu-*CB_1_R*-KO (21.3±2.5%, x^2^ = 0.4189, p = 0.5175) mice. This value practically disappears in *CB_1_R*-KO mice (2.9±2.9%, x^2^ = 15.47, p<0.0001). A similar area was analyzed for each animal condition (1,352 µm^2^ in *CB_1_R*-WT; 1,547 µm^2^ in Glu-*CB_1_R*-KO and 1,274 µm^2^ in *CB_1_R*-KO mice).

The proportion of CB_1_R immunopositive synaptic terminals/preterminals in *CB_1_R*-WT (20.5%) was maintained in Glu-*CB_1_R*-KO mice (20.8%) and reduced in the VMH of GABA-*CB_1_R*-KO mutants (12.4%) (Fig. 4A). CB_1_R immunoparticles virtually disappeared in the VMH of *CB_1_R*-KO mice (Fig. 4A). Furthermore, CB_1_R density in WT and both mutant animals was estimated to be rather low (between 0.40–0.50 immunoparticles/ µm membrane, differences not statistically significant) (Fig. 4B).

We next semiquantified the CB_1_R immunolabeled excitatory axonal boutons to determine the contribution of cortical axons to the pattern of CB_1_R in the VMH. For this purpose, only typical excitatory terminals with abundant clear and spherical vesicles, forming asymmetric synapses with thick postsynaptic densities were taken into account. In this case, 21.3±2.5% and 27.2±0.7% of the asymmetric synapses were CB_1_R immunopositive in the VMH of Glu-*CB_1_R*-KO and *CB_1_R*-WT mice, respectively (Fig. 4C). However, this difference was not statistically significant (x^2^ = 0.4189, p = 0.5175). Finally, the percentage of CB_1_R immunolabeled asymmetric synapses was very low in *CB_1_R*-KO mice (Fig. 4C).

Taken together, these observations indicate that CB_1_R is localized in GABAergic as well as in cortical and subcortical glutamatergic inputs to the VMH.

## Discussion

### CB_1_R is localized in excitatory and inhibitory presynaptic boutons in the VMH

The main finding of this study was the localization of CB_1_R in VMH presynaptic terminals impinging on postsynaptic dendrites and spines of *CB_1_R*-WT, Glu-*CB_1_R*-KO and GABA-*CB_1_R*-KO mice. Furthermore, an extensive analysis of the proportion of immunolabeled profiles identified the contribution of CB_1_R to GABAergic and cortical and subcortical glutamatergic inputs to the VMH.

The dense network of synaptic connections constitutes the anatomical basis for the neuroendocrine and vegetative functions regulated by the hypothalamus. The proportion of CB_1_R immunolabeled synaptic terminals in the VMH of mice lacking CB_1_R in neurons of cortical origin (Glu-*CB_1_R*-KO) was identical to WT animals (∼20%), indicating that CB_1_R probably was in excitatory synaptic terminals of intrinsic hypothalamic neurons. However, although the difference was not statistically significant, the analysis of synaptic terminals forming asymmetric synapses showed a slight decrease of glutamatergic synaptic profiles with CB_1_R in Glu-*CB_1_R*-KO compared to *CB_1_R*-WT mice. Altogether, these results indicate that CB_1_R localizes mostly in subcortical excitatory axon terminals [8], [22], [30], [31] and to a lesser extent in excitatory synaptic boutons of cortical origin [1], [8], [27], [31].

The absence of CB_1_R in forebrain GABAergic neurons (GABA-*CB_1_R*-KO) caused a reduction of the CB_1_R immunolabeled synaptic terminals (12.4%) indicating that CB_1_ receptors are also a molecular component of the GABAergic axon boutons in the VMH. For GABA-*CB_1_R*-KO mutants, DLX mice lead also to recombination in hypothalamic dopaminergic neurons [32]. However, it is unlikely the presence of CB_1_R in dopaminergic synaptic terminals in the VMH of the GABA-*CB_1_R*-KO mutants as there is no tyrosine hydroxylase immunoreactivity in the VMH [32]. Overall, our findings can be interpreted as for the presence of CB_1_R in GABAergic presynaptic terminals of both VMH and intrinsic hypothalamic inhibitory pathways.

### Functional significance

This investigation has demonstrated that CB_1_ receptors in GABAergic and glutamatergic afferents explain the CB_1_R pattern in the VMH. The density of CB_1_R immunoparticles was rather low in GABAergic and glutamatergic boutons in the VMH (∼0.40–0.50 particles/ µm) as compared to the density found in other brain regions [33], [34], particularly in inhibitory synaptic terminals [35]. However, CB_1_R efficiency in the activation of GTP-binding proteins appears to be much higher in the hypothalamus than in other brain regions [14], which may have a functional significance. Physiologically, the identification of CB_1_R in glutamatergic and GABAergic synaptic terminals in the VMH could be regarded as a potential neuronal substrate for the effects of cannabinoids on eating behaviors. Actually, Glu-*CB_1_R*-KO conditional mice exhibit a hypophagic behavior after food deprivation very similar to the full *CB_1_R*-KO. On the contrary, GABA-*CB_1_R*-KO mutants are hyperphagic under the same experimental conditions [26].

As a conclusion, the VMH may be a good hub candidate in the endocannabinoid-mediated modulation of the excitatory and inhibitory neurotransmission regulating food intake behaviors. These anatomical data contribute to the understanding of the complex regulation of energy balance by the endocannabinoid system.

## Materials and Methods

### Ethics Statement

The protocols for animal care and use were approved by the appropriate Committee at the Basque Country University (CEBA/93/2010/GRANDESMORENO). Furthermore, the animal experimental procedures were carried out in accordance with the European Communities Council Directive of 22 July 2003 (2003/65/CE) and current Spanish regulations (Real Decreto 1201/2005, BOE 21–10–2005). Great efforts were made in order to minimize the number and suffering of the animals used.

### CB_1_R mutant lines

Mutant animals were obtained and genotyped as previously described [26], [27], [29]. ***CB_1_R*** **-KO mice** were generated and genotyped as described [29]. Conditional CB_1_R mutant mice were obtained by crossing the respective Cre-expressing mouse line with *CB1* ^f/f^ mice [36], using a three-step breeding protocol [27].

#### Generation of *CB1* ^f/f; NEX-Cre^ mice (here Glu-*CB_1_R*-KO)

*CB1* ^f/f; NEX-Cre^ mice were obtained by crossing *CB1* ^f/f^ with NEX-Cre mice [37], [38]. The helix-loop-helix transcription factor NEX is a marker of embryonic neuronal progenitors, which will develop into mature cortical glutamatergic neurons [39], whereas, in the adult brain, NEX is expressed in mature glutamatergic cortical neurons, but not in cortical GABAergic interneurons and to a much lesser extent in subcortical regions [40]. Cre expression under the control of the regulatory sequences of NEX in transgenic mice (NEX-Cre mice) as generated by knock-in into the NEX locus, leads to the specific deletion of “floxed” alleles in forebrain neurons [37].

#### Generation of *CB1* ^f/f; Dlx5/6-Cre^ mice (here GABA-*CB_1_R*-KO)

Transgenic mice (Dlx5/6-Cre) were produced as previously described [41]. Dlx5/6-Cre mice were crossed with *CB1* ^f/f^ mice to obtain *CB1* ^f/f; Dlx5/6-Cre^ mice [27]. *Dlx5/Dlx6* genes are homeobox genes that are expressed in differentiating and migrating forebrain GABAergic neurons during embryonic development [42]. Thus, expression of Cre recombinase under the control of the regulatory sequences of *Dlx5/Dlx6* genes is expected to drive recombination of loxP sites in GABAergic neurons [27].

### Animal treatment

12 wild-type, Glu-*CB_1_R*-KO, GABA-*CB_1_R*-KO and *CB_1_R*-KO mice (3 of each condition) were used in this study. Mice were deeply anesthetized by intraperitoneal injection of ketamine/xylazine (80/10 mg/kg body weight) and were transcardially perfused at room temperature (RT, 20–25°C) with phosphate-buffered saline (PBS 0.1M, pH 7.4) for 20 seconds, followed by the fixative solution made up of 4% formaldehyde (freshly depolymerized from paraformaldehyde), 0.2% picric acid and 0.1% glutaraldehyde in phosphate buffer (PB 0.1 M, pH 7.4) for 10–15 minutes. Then, brains were removed from the skull and postfixed in the fixative solution for approximately one week at 4°C. Afterwards, brains were stored at 4°C in 1∶10 diluted fixative solution until used.

### CB_1_R immunocytochemistry for light microscopy

Coronal hypothalamic sections were cut at 50 µm in a vibratome and collected in 0.1 M PB at RT. Sections were preincubated in a blocking solution of 10% bovine serum albumin (BSA), 0.1% sodium azide and 0.5% triton X-100 prepared in Tris-HCl buffered saline (TBS 1X, pH 7.4) for 30 minutes at RT. Then, they were incubated in a primary polyclonal goat anti-CB_1_R antibody (2 µg/ml, Frontier Science co. Ltd, 1–777–12, Shinko-nishi, Ishikari, Hokkaido, Japan) prepared in the blocking solution, on a shaker for 2 days at 4°C. The CB_1_R antibody recognizes 31 aminoacids of the C-terminus part (NM007726) of the mouse CB_1_R. After several washes in 1% BSA and 0.5% triton X-100 in TBS, tissue sections were incubated in a secondary biotinylated horse anti-goat IgG (1∶200, Vector Laboratories, Burlingame, CA, USA) prepared in the washing solution for 1 hour on a shaker at RT. The VMH sections were washed in the washing solution described above and processed by a conventional avidin-biotin peroxidase complex method (ABC, Elite, Vector Laboratories, Burlingame, CA, USA). Tissue was incubated in the avidin-biotin complex (1∶50) prepared in the washing solution for 1 hour at RT. Then, sections were washed and incubated with 0.05% diaminobenzidine in 0.1 M PB with 0.5% triton-X100 and using 0.01% hydrogen peroxide as a cromogen, for 5 minutes at RT. Finally, tissue was mounted, dehydrated in graded alcohols (50°, 70°, 96°, 100°) to xylol and coverslipped with DPX. Sections were observed and photographed with a light microscope Zeiss Axiophot. Figure compositions were made at 300 dots per inch (dpi). Labeling and minor adjustments in contrast and brightness were made using Adobe Photoshop (CS, Adobe Systems, San Jose, CA, USA).

### Preembedding immunogold method for electron microscopy

Coronal hypothalamic vibrosections were cut at 50 µm and collected in 0.1 M PB at RT. Sections were preincubated in a blocking solution of 10% BSA, 0.1% sodium azide and 0.02% saponin in TBS for 30 minutes at RT. Then, they were incubated in a primary polyclonal goat anti-CB_1_R antibody (2 µg/ml, Frontier Science co. Ltd, 1–777–12, Shinko-nishi, Ishikari, Hokkaido, Japan) prepared in the blocking solution but with 0.04% saponin, on a shaker for 2 days at 4°C. After several washes with 1% BSA in TBS, tissue sections were incubated in a secondary 1.4 nm nano-gold anti-goat antibody (1∶100, Fab' fragment, Nanoprobes Inc., Yaphank, NY, USA) prepared in the same solution as the primary antibody for 3 hours on a shaker at RT. Then, tissue was washed overnight at 4°C and postfixed in 1% glutaraldehyde for 10 minutes. After several washes in double distilled water, gold particles were silver-intensified with a HQ Silver Kit (Nanoprobes Inc., Yaphank, NY, USA) for 12 minutes in the dark. Then, tissue was extensively washed in double distilled water and in 0.1 M PB and osmicated in 1% osmium tetroxide for 20 minutes. After washing in 0.1 M PB, sections were dehydrated in graded alcohols (50°, 70°, 96°, 100°) to propylene oxide and embedded in Epon resin 812. 80 nm ultrathin sections were collected on mesh nickel grids, stained with lead citrate for 20 minutes and examined in a PHILIPS EM208S electron microscope. Tissue preparations were photographed by using a digital camera coupled to the electron microscope. Figure compositions were made at 300 dots per inch (dpi). Labeling and minor adjustments in contrast and brightness were made using Adobe Photoshop (CS, Adobe Systems, San Jose, CA, USA).

Specificity of the immunostainings was assessed by incubation of the CB_1_R antibody in *CB_1_R*-KO VMH tissue in the same conditions as above.

### Statistical analysis of CB_1_R in the VMH

50-µm-thick hypothalamic sections from each animal condition (n = 3 each) showing good and reproducible silver-intensified gold particles were cut at 80 nm. Image-J (version 1.43 µ, NIH, USA) was used to measure the membrane length. Electron micrographs (18,000–28,000X) were taken from grids (132 µm side) containing silver-intensified gold particles; all of them showed a similar labeling intensity indicating that selected areas were at the same depth. Furthermore, to avoid false negatives, only ultrathin sections in the first 1.5 µm from the surface of the tissue block were examined. Positive labeling was considered if at least one immunoparticle was within approximately 30 nm from the plasmalemma. Metal particles on synaptic membranes were visualized and counted.

Percentages of CB_1_R positive profiles and density of immunoparticles were analyzed and displayed as mean ± S.E.M. using a statistical software package (GraphPad Prism 4, GraphPad Software Inc, San Diego, USA). Group differences were compared by chi-square test, p<0.05 (percentages of CB_1_R positive profiles) and Mann Whitney test, p<0.05 (CB_1_R density).
